# The relationship between lysine 4 on histone H3 methylation levels of alcohol tolerance genes and changes of ethanol tolerance in *Saccharomyces cerevisiae*

**DOI:** 10.1111/1751-7915.12121

**Published:** 2014-04-30

**Authors:** Hang Wang, Binfeng Ji, Hongzhen Ren, Chun Meng

**Affiliations:** Department of Bioengineering, College of Biological Science and Biotechnology, Fuzhou UniversityFuzhou, Fujian, China

## Abstract

We evaluated whether epigenetic changes contributed to improve ethanol tolerance in mutant populations of *Saccharomyces cerevisiae* (*S. cerevisiae*). Two ethanol-tolerant variants of *S. cerevisiae* were used to evaluate the genetic stability in the process of stress-free passage cultures. We found that acquired ethanol tolerance was lost and transcription level of some genes (*HSP104*, *PRO1*, *TPS1*, and *SOD1*) closely related to ethanol tolerance decreased significantly after the 10th passage in ethanol-free medium. Tri-methylation of lysine 4 on histone H3 (H3K4) enhanced at the promoter of *HSP104*, *PRO1*, *TPS1* and *SOD1* in ethanol-tolerant variants of *S. cerevisiae* was also diminished after tenth passage in stress-free cultures. The ethanol tolerance was reacquired when exogenous *SOD1* transferred in some tolerance-lost strains. This showed that H3K4 methylation is involved in phenotypic variation with regard to ethanol tolerance with respect to classic breeding methods used in yeast.

## Introduction

Ethanol is one of the oldest biochemical products known to human civilization. It has been widely used for human consumption and as an industrial chemical and fuel. *Saccharomyces cerevisiae*, the brewers' (budding) yeast, is the primary microorganism used in the production of ethanol through fermentation.

Yeast strains with good tolerance to high concentrations of ethanol are highly desirable. Recently, some modern genetic approaches, such as global transcription machinery engineering (Lam *et al*., 2010; Lanza and Alper, 2011), transposon mutagenesis (Kim *et al*., 2011) and genome shuffling (Hou, 2010; Liu *et al*., 2011; Pinel *et al*., 2011) have been developed to improve ethanol fermentation performance of *S. cerevisiae*. However, it is still difficult to obtain such strains through modern genetic modification because ethanol tolerance to high concentration of alcohol is a very complex phenotype, involving the expression of many genes. More than 250 genes are believed to be involved in ethanol tolerance (Hu *et al*., 2007; Auesukaree *et al*., 2009; Teixeira *et al*., 2009; Hou, 2010; Mira *et al*., 2010).

Although time-consuming, laborious and inefficient, classical mutagenesis methods of treating organisms with physical irradiation or chemical mutagens are one of the main ways of improving microorganism strains with regard to environmental tolerance (Patnaik *et al*., 2002; Stephanopoulos, 2002; Zhang *et al*., 2002; Pereira *et al*., 2003; Rosenfeld *et al*., 2003; Lam *et al*., 2010; Mira *et al*., 2010; Zhao *et al*., 2010; Fiedurek *et al*., 2011; Yang *et al*., 2011; Kumari and Pramanik, 2012; Tao *et al*., 2012; Kim *et al*., 2013; Wang *et al*., 2013). Simultaneous improvements of these related genes in cells have proven to be difficult through the molecular biological methods because of a lack of the necessary genetic knowledge and tools for genetic modification on the multiple-gene level.

We have found that deterioration of desired traits in production strains often occurs during serial passage cultures when the screening pressure for the desired function is relaxed, and this also occurs during long-storage periods and at low temperatures. We also found that regressive traits can be obtained after continuous culture with a selecting pressure. Genetic mutagenesis of microbes is considered the main cause of trait deterioration and trait loss (Glazer *et al*., 1991; Mortimer *et al*., 1994; Kolodner *et al*., 2002; Piazza *et al*., 2010). The observation that desired traits are easily lost under relaxed selection pressures and recovered under screening medium in the presence of that selection pressure is no longer surprising, but the specific genetic mechanism responsible for both loss and recovery remains elusive.

To obtain desirable traits, corresponding metabolic pathways are often regulated through increase or reduction of the expression of specific genes on the transcriptional level. Not mutagenesis but epigenetic changes of some genes might be the means of affecting gene expression.

Epigenetic phenomena include DNA methylation, histone modification and chromatin remodelling. Covalent modifications of histones, which induce to remodel chromatin, also produce heritable phenotypes independently of alterations in gene sequence. In eukaryotic cells, DNA methylation, histone modification and chromatin remodelling induced trait loss and recovery, especially in fungi. Fungal epigenetic modifications are known to be established and modified in response to environmental factors (Waterland and Jirtle, 2003; Jablonka and Raz, 2009; Patalano *et al*., 2012). Methylation and acetylation are the most highly studied of these epigenetic changes. For example, methylation of H3K4 and of H3K9 by histone methyltransferases and acetylation of the histone H3K4 and H3K9 by histone acetyltransferase enzymes is generally correlated with transcriptional competence in yeast strains (Pokholok *et al*., 2005). In this study, we investigated the H3K4 tri-methylation change in certain target genes in different yeast strains.

## Results

### Ethanol-tolerance phenotype stability of *S. cerevisiae*

**Fig. 1:**
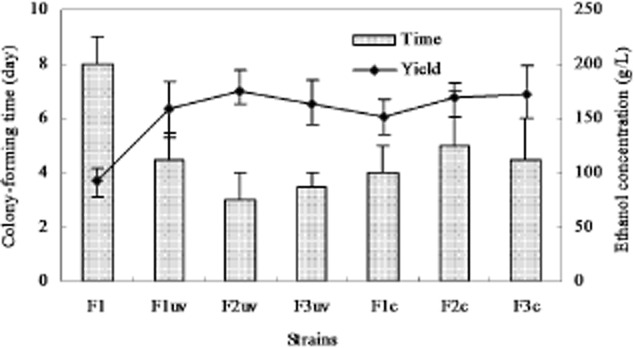
The colony-forming time in the plate contained ethanol and ethanol yield of strain F1 or F1 after treatment with UV or breeding through the acclimation. The times at which strains formed colonies were used to evaluate their tolerance and as the main criterion for the strain screening. The ethanol yield was performed in a fed-batch fermentation process with a 5 L fermenter. The final ethanol concentrations in the media were detected after 50 h of fermentation.

We used two classical breeding methods to select populations of ethanol-tolerant variants from wild-type *S. cerevisiae* F1. One ethanol-tolerant population was selected from a UV mutant library cultured on plates containing 150 mg ml^−1^ of ethanol. The other ethanol-tolerant population was obtained by chemostat-mediated acclimation of *S. cerevisiae* F1 to high-ethanol concentrations in a 500 ml fermenter in which the ethanol concentration was slowly increased from 80 mg ml^−1^ to 150 mg ml^−1^ over a period of 800 h. Figure 1 shows a comparison of the wild-type strain F1 and the mutant variant strains Fuv1, Fuv2 and Fuv3, selected from the UV mutation batch, and F1c, F2c and F3c, obtained from the chemostat-mediated acclimation batch. The breeding populations were noticeably improved with regard to ethanol tolerance and production relative to wild type.

**Fig. 2:**
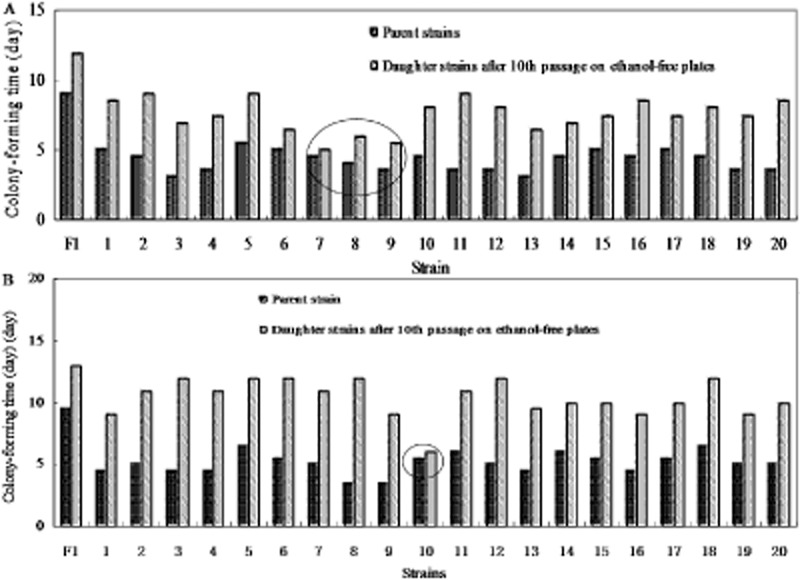
The colony-forming time difference between parent strains and their relative daughter strains obtained through serial-passage cultures on ethanol-free plates, which all the strains cultured on solid media plates containing about 150 g L^−1^ of ethanol. A. Parent strains screened from UV-treated strains. B. Parent strains obtained through chemostat acclimation. In order to evaluate their tolerance to high ethanol, yeast cells were incubated on solid agar media containing 150 g L^−1^ ethanol sealed with double plastic bags at temperature of 30°C. The time that strains took to form colonies was the parameter to evaluate its tolerance abilities to ethanol.

To assess the resistance phenotype stability of the two improved populations, we selected 20 strains from the UV mutant library and chemostat-mediated acclimation library, respectively, and performed serial-passage cultures on ethanol-free plates. After the 10th passage, these daughter strains and their parent strains were cultured on solid media plates containing about 150 g L^−1^ of ethanol. The colony-forming time is used for evaluating growth difference between the parent strain and its daughter strains. Growth results showed that most of the 40 strains reduced ethanol tolerance and showed considerable ethanol-tolerant phenotypic instability and their colony-forming time extended 2 more days than the original strains, with exception that three mutant strains (strain 7, 8, 9) from UV mutant library (Fig. 2A) and one mutant strain (strain 10) from chemostat-mediated acclimation library (Fig. 2B) maintained the ethanol tolerance with no significant differences of colony-forming time between daughter strains and parent strains (the strains marked with circle in Fig. 2).

### Changes of H3K4 methylation level at the promoter regions

Because brewers' yeast (*S. cerevisiae*) undergoes low level DNA methylation (Selker *et al*., 2003), we focused on the relationship between genetic instability with regard to ethanol tolerance and variations in histone lysine methylation on selected four ethanol-tolerant key target genes.

In *S. cerevisiae*, histone lysine methylation has been shown to occur on lysine residues 4, 9, 36 and 79 of histone H3 (H3K4, H3K9, H3K36 and H3K79) and to be coupled tightly to the process of transcription (Lee *et al*., 2005; Pokholok *et al*., 2005). Methylation of H3K9 demarcates heterochromatin to silence the gene expression, whereas H3K4 methylation demarcates euchromatin to promote maintenance of active chromatin (Barski *et al*., 2007; Benevolenskaya, 2007; Li *et al*., 2008). To determine whether H3K4 methylation is responsible for the acquired ethanol tolerance, we randomly selected seven ethanol-tolerant mutant strains (*Fuv1*, *Fuv2*, *Fuv3*, *Fuv4*, *Fuv5*, *Fuv6* and *Fuv7*) and their daughters passed 10th passage on ethanol-free plates (Fuv1′, Fuv2′, Fuv3′, Fuv4′, Fuv5′, Fuv6′ and Fuv7′), and seven acclimatized ethanol-tolerant strains (*Fc1*, *Fc2*, *Fc3*, *Fc4*, *Fc5*, *Fc6* and *Fc7*) and their daughters passed 10th passage on ethanol-free plates (Fc1′, Fc2′, Fc3′, Fc4′, Fc5′, Fc6′ and Fc7′) from the 40 strains mentioned above, and then analyzed H3K4me3 and H3K9me3 methylation at the promoter regions of the four target genes, *HSP104* (Heat Shock Protein 104), *PRO1* (encoding γ-glutamyl kinase), *TPS1* (trehalose-6-phosphate synthase 1) and *SOD1* (superoxide dismutase 1) that play important roles in ethanol tolerance.

**Fig. 3:**
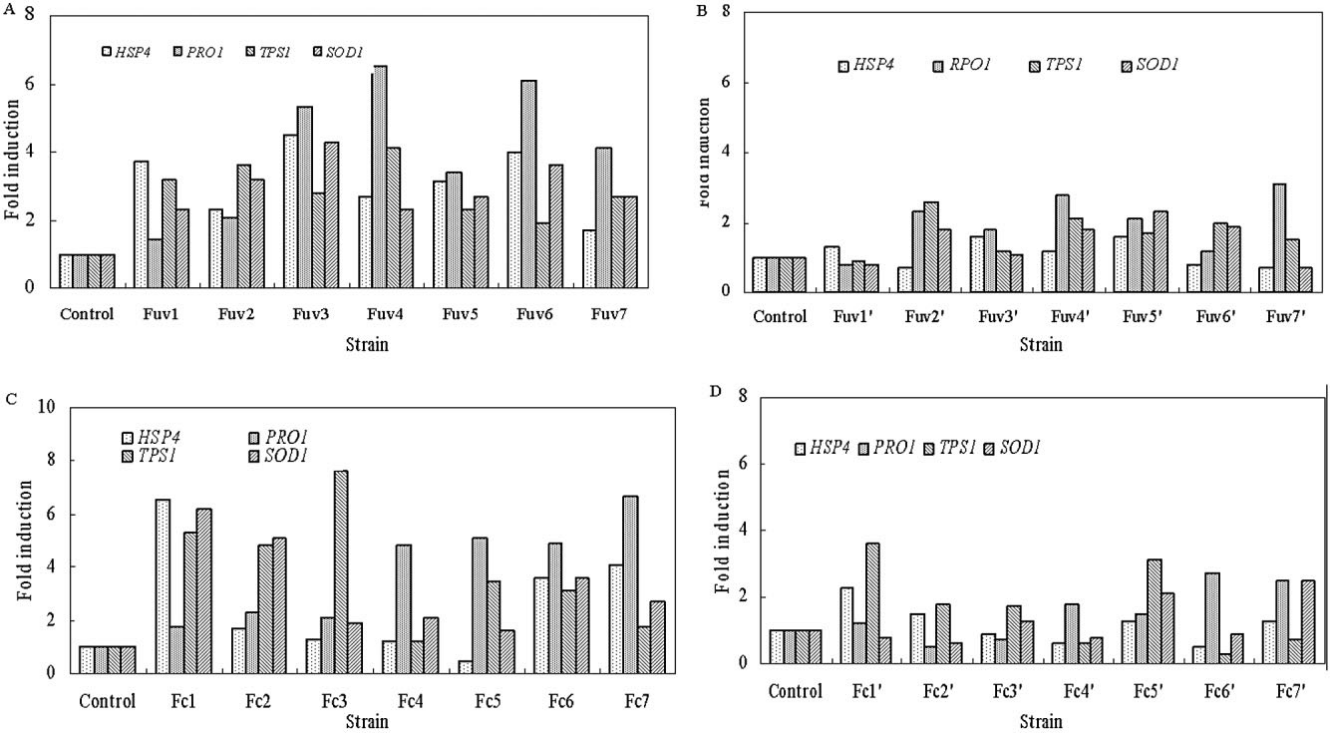
*HSP104*, *PRO1*, *TPS1* and *SOD1* H3K4me3 methylation comparison of mutant variant strains and wild strain. H3K4me3 methylation was analyzed by semi-quantitative PCR at the promoter region of the target genes, *HSP104*, *PRO1*, *TPS1* and *SOD1*. The methylation level is a ratio of the variant mutant strains to the wild-type strain. A. UV-treated strains. B. UV-treated strains after the tenth passage culture. C. Strains obtained from chemostat-mediated acclimation. D. Strains obtained from chemostat-mediated acclimation after the tenth passage culture.

**Fig. 4:**
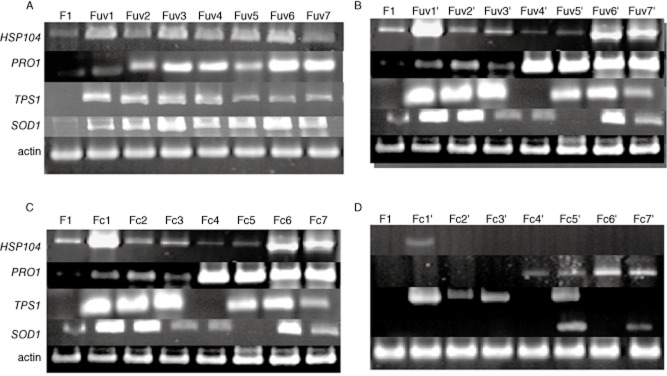
*HSP104*, *PRO1*, *TPS1* and *SOD1* expression levels in the mutant variants and the wild-type strain. Expression levels were analyzed by RT-PCR and actin expression was used as the control. A. UV-treated strains. B. UV-treated strains after the tenth passage culture. C. Strains obtained from hemostat-mediated acclimation. D. Strains obtained from hemostat-mediated acclimation after the tenth passage culture.

No significant differences in H3K9 tri-methylation were observed between the breeding high-yield strains and 10-passage low-yield strains obtained in ethanol-free medium (data not shown). We observed enhanced tri-H3K4 methylation at the promoter region in *HSP104, PRO1, TPS1* and *SOD1* in breeding high-yield strains, while H3K4 methylation level at the promoter region diminished when these cells were performed with passage culture in ethanol-free medium (Fig. 3). We then used reverse transcription polymerase chain reaction (RT-PCR) to analyze the transcriptional levels of the target genes. The results showed that *HSP104*, *PRO1*, *TPS1* and *SOD1* were high activated in breeding high-yield strains while expressed in a low level after passage culture in ethanol-free medium (Fig. 4). This is consistent with the increased H3K4 methylation levels observed. All the four genes showed more than 99.9% sequence similarity between the parental strain and those daughter strains respectively (data not shown).

### Effect of SOD1 expression on the ethanol tolerance of *S. cerevisiae*

**Fig. 5:**
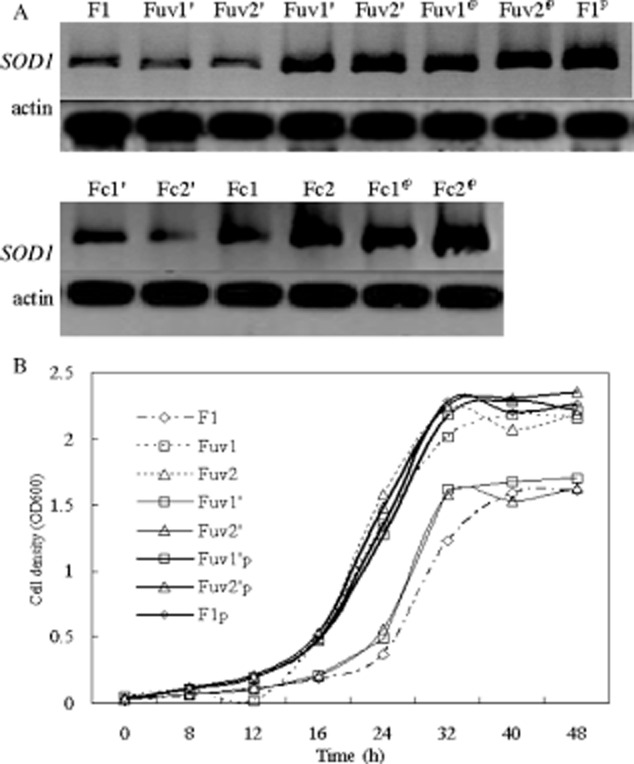
Effect of *SOD1*expression on yeast growth. A. Cells were harvested at OD600 of 1.0 for *SOD1* detection. B. Cells were inoculated in medium supplemented with 10% (v/v) ethanol at an initial OD600 of 0.02. The cultures were incubated at 30°C with aeration for 48 h prior to measuring their optical densities.

To investigate the functional role of these genes, we selected some strains (Fuv1, Fuv2, Fuv1′, Fuv2′, Fc1, Fc2, Fc1′, Fc1′) with obvious changes on *SOD1* expression level to examin whether *SOD1* activity was required to combat the ethanol stress for yeast strains. The plasmid that contained *SOD1* gene was transferred in low-yield strains and wild strains (expressed as F^p^). The *SOD1* Western blotting showed that the transferred *SOD1* was successfully expressed in low-yield strains Fuv1′^p^, Fuv2′^p^, Fc1′^p^, Fc2′^p^ and wild strains F1′^p^ (Fig. 5A). Wild strain F1, Fuv1′, Fuv2′, Fc1′ and Fc1′ showed a longer lag phase and reached saturation at a lower cell density than Fuv1, Fuv2, Fc1 and Fc1. Both wild-type (F1′^p^) and low-yield strains (Fuv1′^p^, Fuv2′^p^, Fc1′^p^ and Fc2′^p^) grew well when *SOD1* gene was transferred in (Fig. 5B). The *SOD1* expression increased the ethanol stress significantly.

## Discussion

The mechanism how ethanol is toxic to yeast cells has not been fully understood, but the previous results showed that the toxicity of ethanol was related to decrease of water availability and chaotropic stress. Lowering water activity mediated by ethanol could interfere with hydrogen bonding within and between hydrated cell components and cell metabolism. Chaotropic stress caused by ethanol might be another major parameter to inhibit microbial metabolism through reducing structural interactions within and between biomacromolecule, changing cellular osmotic pressure, acting as toxic hydrophobic substances in macromolecular and cellular systems, destroying membrane-lipid composition and producing reactive oxygen species in cells.

Some compatible solutes, including trehalose, aliphatic polyols, proline, etc. have been found to protect the microbial cells against the chaotropicity of ethanol effectively in the range of *in vitro* and *in vivo* studies of alcohol tolerance (Mansure *et al*., 1994; Hallsworth *et al*., 2003a,b; Bhaganna *et al*., 2010; Cray *et al*., 2013a,b). So high-level PRO1 (proline synthesis) and TPS1 (trehalose synthesis) is very useful to protect the *S. cerevisiae* against the toxity of ethanol. Hsp104 is required for tolerance to many forms of stress in *S. cerevisiae* through disassembling protein aggregates, which have accumulated in response to stress (Bhaganna *et al*., 2010). Some works have confirmed the role superoxide dismutases-CuZnSOD (encoded by *SOD1*) in the build-up of tolerance to ethanol during growth of S. cerevisiae from exponential to post-diauxic phase. Ethanol toxicity is correlated with the production of reactive oxygen species (free radicals) in *S. cerevisiae* cells. Overexpression of CuZnSOD could not only eliminate free radicals and but also prevent free radicals diffusion to the cytosol, thereby protecting lipids, proteins and nucleic acids from oxidative damage (Bhaganna *et al*., 2010; Bleoanca *et al*., 2013).

These key genes are required for ethanol tolerance in *S. cerevisiae*, which are essential to increase yeast cell tolerance to ethanol and other stress. So keeping or increasing the transcriptional levels of these tolerant-relative genes is a potential method to obtain high-yield strains.

Many examples have demonstrated that epigenetic change play an important role in responses to environmental stimuli through altering the epigenetic state of the genome to influence the appropriate gene expression level in plant cells and animal cells (Prazeres *et al*., 2011; Gudsnuk and Champagne, 2012; Kubota *et al*., 2012). We have also believed that H3K4 methylation might control the metabolic patterns in *S. cerevisiae* cells although there were not many the relative reports about epigenetic control of the fission yeast genome. In the work, we found that tri-methylation of H3K4 level in the histone region of binding the promoters of *HSP104*, *PRO1*, *TPS1* and *SOD1* have influenced their transcription level, which also show that changes in tri-methylation of H3K4 level are correlated with ethanol resistance. In the low ethanol tolerance strains with low-transcriotional level of *SOD1*, the transferred *SOD1* genes increased their ethanol tolerance considerably as a direct result of overexpression of *SOD1*. Effect of increasing of transcription levels of *HSP104*, *PRO1* and *TPS1* on ethanol tolerance ability is worthy of further research.

The tri-methylation of H3K4 analysis of four genes described in this work reveals new information on ethanol resistance mechanisms in yeast. Degenerate of ethanol-tolerant ability after passage on the ethanol-free medium showed high-ethanol concentrations might be required for maintaining ethanol tolerance of *S.cerevisiae*. We always focus on the gene mutation when seeking strong ethanol-tolerant yeast strains. Not enough work has been paid on the effect of ethanol itself on ethanol-tolerant variation of yeast cells. Environmental factors have been also proved to influence epigenetic changes.

The relationship between high-concentration ethanol and high tri-methylation of H3K4 level of the four genes was required to be investigated in the future work. The high-concentration ethanol might contribute to the changes of epigenetic pattern of *S. cerevisiae* as well as the selection of ethanol-tolerant strains, which may also explain the mechanism of classic domestication breeding in yeast in part.

DNA mutations that lead to changes in the gene sequence are considered the main cause of genetic instability. We have shown that the epigenetic mechanisms may also play an important role in phenotypic improvement in industrial strains. Our results explain why acquired traits of industrial strains can be easily lost, which has been a problematic issue in selecting and maintaining industrial strains. How to prevent loss of desirable traits in industrial strains waits for its answer. Epigenetic changes might be an important complement to traditional molecular mechanisms of breeding.

## Conclusion

Together, these results indicate significant changes in epigenetic factors in mutant cells, suggesting that, in addition to DNA sequence mutations, other factors also play an important role in trait improvement in breeding strains. This result indicated that ethanol tolerance was likely acquired through epigenetic changes rather than DNA mutation. The traits acquired through DNA mutation should not disappear quickly because the natural spontaneous mutation rate is not always high and specific, whereas the traits acquired through H3K4 tri-methylation change often show instability along with the changes of the environment.

## Experimental procedures

### Strains and media

We used a standard laboratory strain of wild *S. cerevisiae* F1 for *S. cerevisiae* cultivation (a strain selected from the American Type Culture Collection 28097 Haploid), yeast extract peptone dextrose medium consisting of 10 g L^−1^ of yeast extract (OXLP0021B, Thermo Scientific, Shanghai, China), 20 g L^−1^ of Bacto Peptone (BD DIFCO, NJ, USA), and 20 g L^−1^ of glucose (Edible, Shandong Xiwang, Shandong, China) were used, with an adjusted pH value of 6.0. For ethanol production in flasks, fermentation medium containing 250 g L^−1^ of glucose, 4 g L^−1^ of yeast extract, 0.5 g L^−1^ of (NH4)_2_SO_4_, and 2 g L^−1^ of KH_2_PO_4_ with an adjusted pH value of 6.0 were used. (The glucose concentrations were adjusted according to actual demand in bioreactor fermentation.)

### UV mutagenesis and tolerance to ethanol stresses

Mutagenesis was carried out using UV irradiation. Cells at a concentration of 10^6^–10^8^ ml^−1^ were irradiated under a 30W UV light at a distance of 25 cm for 50 s and then treated with 1% dimethyl sulfate for 2 min at room temperature.

For selection of the ethanol tolerance phenotype, a yeast library was initially placed on solid media containing 100 g L^−1^ of glucose and containing about 150 g L^−1^ of ethanol. For the stress experiments, 160 g L^−1^ of ethanol was added when autoclaved media were cooled to about 45°C. Then the plates contained ethanol were sealed with double plastic bags to minimize the risk of volatilization. Each culture plate sealed with double plastic bags was incubated at 30°C. There are about 0.85 ± 0.21 g L^−1^ and 0.62 ± 0.26 g L^−1^ of ethanol lost in the cooling process and the culturing process respectively. The time that a strain took to form megascopic round, smooth, white colonies under the selected condition was the primary parameter to evaluate its tolerance to the selected ethanol concentration, and thus was used as the main criteria for the strain screening.

### Evaluation of fermentation via bioreactor

The volumes of ethanol produced and detected in the media compare with those of wild-type yeast cultured under the same conditions. Characterization of anaerobic fermentation for different strains was carried out in a B. Braun5 fermenter (Sartorius AG, Weender Landstr, Goettingen, Germany). Fermentation was initiated by inoculating 300 ml of overnight culture into 2 L medium (pH 6.0). The fermenter was kept at 30°C with an agitation speed of 100 r.p.m., and the pH values of the cultures were monitored and recorded automatically throughout. The cultures were constantly fed 60% (w/v) glucose to maintain a stable glucose concentration of 100 g L^−1^. Samples were periodically drawn from the reactors and monitored for ethanol and cell concentrations.

Ethanol was assessed via gas chromatograph (GC, Varian model 3700, Varian Inc., Palo Alto, CA, USA) equipped with a flame ionization detector (Varian, Inc.) and auto linear temperature programmer (Varian, Inc.). The carrier gas was nitrogen. The column was packed with 1–1814 80/120 Carbopack B/6.6% Carbowax 20 M (Supelco, Sigma-Aldrich, Bellefonte, PA, USA).

### Determination of messenger RNA (mRNA) expression

Total RNA was isolated from relative yeast cells cultured in the ethanol-free liquid medium using the QuickPrep RNA extraction kit (ABi, Carlsbad, CA, USA) according to manufacturer's protocol. Complementary DNA (cDNA) was synthesized from 0.5 μg of RNA using the first strand cDNA synthesis kit (MBI Fermentas, Vilnius, Lithuania). Primers were synthesized by the DNA Synthesis Centre, Sangong, China. The primers used to analyze the expression levels of *HSP104*, *PRO1*, *TPS1* and *SOD1* were as follows (Table 1). mRNA levels of various genes were determined by SYBR Green I semiquantitative PCR according to Abi 7300 protocol described (Life Technologies, Carlsbad, CA, USA). All mRNA levels were normalized to β-actin mRNA. Normalization to β-actin mRNA was found to give comparable results (Schmittgen and Livak, 2008).


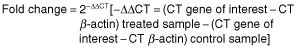


This form of the equation was used to compare the gene expression in the treated sample and the untreated control.

**Table 1:** Primers used for PCR in this study

Primer name	Forward (5′→3′)	Reverse (5′→3′)
β-actin	GGTCTCAAACATGATCTGGG	GGGTCAGAAGGACTCCTATG
HSP104 Promoter	TATATCACAGTAAAAGGCAAAGGGG	CTGATTCGATTCAAGGGTAA
PRO1 Promoter	CATTAAGCATGTTTTG	TTTTAACGGATCACAA
TPS1 Promoter	GGGCCTATACGGTGAA	ACCCGATGCAAATGAG
SOD1 Promoter	CGCTACAGACAGGCGTTAA	ACCCGATGCAAATGAGAC
HSP104	GTTTCCAATGCCGTTAGATTGTCTA	GACCGCATACTTCTCG
PRO1	GCTATTGGGCAGGGTA	TGGCATCTGGGTTTGT
TPS1	AAGCAGGCTAACAAAC	TCAGGAAGATGGGTAC
SOD1	AGCAGTCGCAGTGTTA	AGTGAGGACCAGCAGA

### Construction of SOD1 expression vector

Genomic DNA was extracted from the *S. cerevisiae* cells using a Wizar Genomic DNA Purification Kit, according to the manufacturer's instructions (Promega, Shanghai Promega Ltd., Shanghai, China). The genomic DNA was then used as a template for PCR. The primers used for amplification of a genomic DNA encoding the SOD1 were 5′-CCACTCGAGATGGTTCAAGCAGTCG-3′ for the translational start sequence region and 5′-CGACCGCGG AAAA GA AAAA GA CATTAACATTAGTTGATTAGA-3′ for the 3′terminator region (The underline bases were the restriction enzyme cutting site). After a 35-cycle amplification (94°C for 30 s, 50°C for 40 s, 72°C for 2 min), PCR products were analyzed with 1.0% agarose gel electrophoresis. The PCR product then was purified with a PCR Purification Kit (QIAGEN, Hilden, Germany) and cloned into the plasmid vector PICZαA after digested with restriction endonuclease *Xho*I and *Sac*II respectively.

### Chromatin immunoprecipitation (ChIP) assay

ChIP products were analyzed by quantitative real-time PCR using SYBR green real-time PCR with an ABi7300 iCycler as previously described (Zhao *et al*., 2010; Fiedurek *et al*., 2011; Yang *et al*., 2011). The initial strains and the 10th passage strains harvested from liquid culture for 24 h were fixed, lysed and sonicated respectively. Sonicated lysates equivalent to 8 × 106 99 cells were subjected to ChIP analysis. The comparative CT method was used to determine relative expression compared with input, which was then aver100 aged over three independent experiments. In this experiment, the H3k4 and H3K9 trimethylation antibodies were purchased from Upstate (Upstate Biotechnology, Inc., Lake Placid, New York, USA) and Abcam (Abcam, Inc. Cambridge, MA, USA).
